# Role of age in presentation, response to therapy and outcome of autoimmune hepatitis

**DOI:** 10.1038/s41424-018-0028-1

**Published:** 2018-07-02

**Authors:** Martine A. M. C. Baven-Pronk, Maaike Biewenga, Joanne J. van Silfhout, Aad P. van den Berg, Henk R. van Buuren, Bart J. Verwer, Carin M. J. van Nieuwkerk, Gerd Bouma, Bart van Hoek

**Affiliations:** 1 Department of Gastroenterology and Hepatology, Leiden University Medical Center, Leiden, The Netherlands; 2 Department of Gastroenterology and Hepatology, Green Heart Hospital, Gouda, The Netherlands; 3 Department of Gastroenterology and Hepatology, University Medical Center Groningen, Groningen, The Netherlands; 4 Department of Gastroenterology and Hepatology, Erasmus University Medical Center, Rotterdam, The Netherlands; 5 Department of Gastroenterology and Hepatology, Vrije University Medical Center, Amsterdam, The Netherlands

## Abstract

**Background:**

Few studies with diverging results and a small sample size have compared autoimmune hepatitis (AIH) in the elderly to younger patients.

**Aim:**

To unbiasedly investigate the role of age in behaviour and treatment outcome of AIH.

**Methods:**

All patients with probable or definite AIH type 1 in four tertiary academic centres were included in this retrospective—and since 2006 prospective—cohort study. Influence of age on presentation, remission and outcome of AIH were investigated.

**Results:**

359 patients were included. Presence of cirrhosis at AIH diagnosis around 30% was independent of age. ALAT was higher at age 30–60 years on AIH diagnosis, and above age 60 there were less acute onset, less jaundice and more concurrent autoimmune disease. Remission was reached in 80.2%, incomplete remission in 18.7%, only 1.1% (all aged 50–65) was treatment-refractory. Age was not an independent predictor of remission, while cirrhosis was. Above age 45 there was more diabetes, above age 60 more loss of remission. Rate of progression to cirrhosis was 10% in the 10 years after diagnosis and unrelated to age at AIH diagnosis. With onset below age 30, there was more development of decompensated cirrhosis over time. With higher age at AIH diagnosis there was a lower survival free of liver-related death or liver transplantation.

**Conclusions:**

AIH presents at all ages. Age influences features at diagnosis, but not response to treatment, while survival without liver-related death or liver transplantation decreases with higher age at diagnosis.

## Introduction

Autoimmune hepatitis (AIH) is a chronic progressive inflammatory liver disease responsive to immunosuppression^1,2^. Originally AIH was believed to be a disease of young women^3,4^. Currently it is known that AIH can present at all ages. Several studies indicate an incidence pattern with two age peaks, one in the second decade and one between the fourth and sixth decade^5–9^. Others show a single peak between the fourth and seventh decade^10–12^.

Ten studies with relatively small sample size have specifically addressed AIH in elderly patients with an arbitrary age cut-off at 60 or 65 years and have yielded diverging results^5,6,8–15^. These data were recently included in a meta-analysis, which concluded 20%–25% of patients are above the age of 60 at diagnosis, that patients above 60 years of age were more likely to be cirrhotic and asymptomatic at diagnosis, had the same response to treatment as compared to younger patients, but were less likely to relapse after withdrawal of treatment^16^. The aim of this multicentre, retrospective, observational study was to unbiasedly investigate the role of age at diagnosis regarding presentation, response to therapy and outcome in a large group of patients with AIH type 1.

## Patients and methods

All patients diagnosed with probable of definite AIH according to the International AIH group (IAIHG) criteria from four academic centres were included^1^. Since August 2006 all previously known and new patients are prospectively included in a national database. All patients with anti-LKM antibodies—which were only present in the younger group, presumably with AIH type 2, which has a different clinical course—and patients with overlap syndromes, as defined by the Paris criteria for PBC and by cholangiography criteria for PSC, were excluded^2,17,18^.

Informed consent was obtained from each patient included in the study and the study protocol conforms to the ethical guidelines of latest revision of the 1975 Declaration of Helsinki as reflected in a priori approval by the institution’s human research committee.

Data concerning mode of presentation, baseline clinical, laboratory and liver histological characteristics, concomitant autoimmune disease, results and adverse effects of immunosuppressive treatment and long-term prognosis, were retrospectively retrieved by chart review. The mode of onset could be acute (symptom onset to diagnosis less than six months), insidious (symptom onset to diagnosis more than six months), or asymptomatic (no symptoms, AIH accidentally discovered). Response to treatment was defined according to the criteria in the AASLD guideline^19^. Treatment failure was defined as: worsening of clinical, laboratory and—if available—histological features of interface hepatitis despite compliance with therapy. Incomplete response was defined as some improvement in clinical laboratory without normalisation of serum aminotransferases and—if available—histological presence of interface hepatitis despite compliance with therapy. Remission was defined as disappearance of symptoms, normal serum aminotransferases, bilirubin and IgG—and if histology was available—no interface hepatitis or normal hepatic tissue or inactive cirrhosis; Loss of remission was defined as an increase in serum aminotransferase levels above the upper limit of normal on at least two occasions after having been in remission with or without clinical symptoms and the need to adjust or reinstitute drug therapy^20^. Relapse was defined as serum aminotransferase levels of more than threefold the upper limit of normal after having been in remission. Decompensated cirrhosis was defined as presence of ascites, hepatic encephalopathy, or oesophageal varices. Duration of follow up was defined as the time between diagnosis and the date of last outpatient appointment, liver transplantation or death.

Primary endpoints were presentation, remission and the combined endpoint of liver-related death or liver transplantation. Secondary endpoints were differences in biochemistry and serology, symptoms, mode of presentation, concurrent autoimmune diseases, initial and maintenance treatment regimens, number of switches of therapy, adverse effects of treatment, episodes of loss of remission, number of relapses and cirrhosis at presentation and disease progression (to cirrhosis, decompensated cirrhosisliver transplantation or death).

Results were reported across all ages and with a 60- and 65-year-cut-off.

For statistical analysis ANOVA, Fisher’s exact test, Chi square test, Mann–Whitney *U* test and independent samples *T* test were used where appropriate. Kaplan–Meier (KM) survival analysis, Cox regression analysis, Poisson distribution and log-rank test were used to correct for the statistically significant differences in follow up. *p* < 0.05 was considered the level of significance.

## Results

### Presentation

**Fig. 1:**
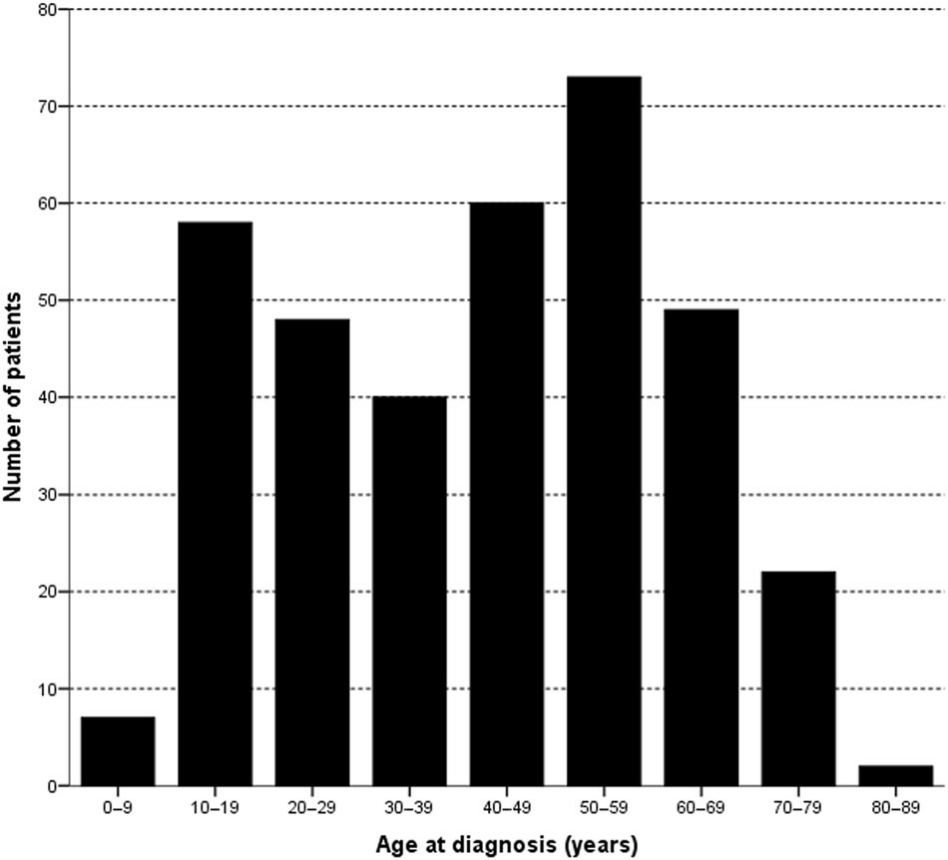
**Distribution of age at diagnosis of AIH in 359 patients with AIH type one**

A total of 359 patients with probable and definite AIH were identified from four academic centres. The distribution of the age at diagnosis showed a bimodal pattern (Fig. 1).

Symptoms and laboratory values per age category are shown in Table 1. There was a similar percentage of cirrhosis (mean 29.7%) at diagnosis of AIH across ages. There were no significant differences across age categories in mode of presentation; nevertheless there was a trend towards less acute presentation with AIH onset above the age of >60 years, more asymptomatic presentation with onset between 40 and 70 years, more insidious presentation between 70 and 79 years and less insidious presentation with onset at 40–49 years. The incidence of HLA-DR4 with onset at or above 40 years vs below 40 years was 35% vs 12.5% (*p* = 0.001). Alanine aminotransferase (ALAT) levels were higher in patients with ages 30–60 years at onset (*p* < 0.001), while alkaline phosphatase (ALP), ALP/ALAT ratio and albumin serum levels were similar across ages categories. International normalised rartio op prothrombin time (INR) was higher with onset below 20 years (*p* < 0.05) and between 40–49 years (*p* < 0.05) of age at onset. There was more jaundice with diagnosis of AIH below 60 vs at/above 60 years (47.7% vs 26.1%, *p* = 0.001). Incidence of fatigue was not different across ages. Frequencies of other symptoms were too low to reliably asses differences across age categories. Histological parameters were not different across age categories (not shown).

**Table 1:** Characteristics on presentation per age category

	Age on diagnosis (year)									
	0–9 (*N* = 7)	10–19 (*N* = 58)	20–29 (*N* = 48)	30–39 (*N* = 40)	40–49 (*N* = 60)	50–59 (*N* = 73)	60–69 (*N* = 49)	70–79 (*N* = 22)	80–89 (*N* = 2)	*p*-value
Cirrhosis on diagnosis	2 (29%)	21 (36%)	10 (21%)	17 (43%)	14 (230%)	17 (230%)	15 (310%)	8 (38%)	2 (100%)	0.076
Mode of onset										0.401
Asymptomatic	0 (0%)	5 (12%)	7 (18%)	4 (11%)	11 (19%)	11 (16%)	11 (23%)	2 (10%)	1 (50%)	
Insidious	2 (67%)	29 (69%)	20 (53%)	25 (68%)	33 (38%)	42 (61%)	32 (68%)	17 (81%)	0 (0%)	
Acute	1 (33%)	8 (19%)	11 (29%)	8 (22%)	13 (23%)	16 (23%)	4 (9%)	2 (10%)	1 (50%)	
Concurrent autoimmune disease	1 (14%)	10 (17%)	10 (21%)	7 (18%)	15 (25%)	14 (19%)	8 (16%)	6 (27%)	0 (0%)	0.337
HLA DR3	1 (50%)	20 (71%)	17 (68%)	9 (53%)	11 (42%)	30 (65%)	11 (52%)	4 (57%)	0 (0%)	0.422
HLA DR4	0 (0)	1 (4%)	5 (20%)	3 (18%)	10 (39%)	16 (35%)	8 (38%)	1 (14%)	0 (0%)	0.337
ALAT (IU/l)	231	379	454	422	441	663	273	319	266	**0.001**
ALP (IU/l)	360	197	138	151	140	172	138	146	111	0.134
ALP/ALAT ratio	1.885	0.520	0.393	0.490	0.300	0.240	0.635	0.515	0.406	0.099
Albumin (g/l)	33.0	38.0	37.5	38.5	38.0	39.0	36.0	37.0	36.0	0.787
INR	10.5	11.9	1.9	1.6	6.6	1.2	1.3	6.7	1.0	0.598
IgG (g/l)	51.6	25.1	22.8	23.1	25.0	21.6	22.9	28.6	20.2	**0.010**
Symptoms^a^										
Jaundice	1 (33%)	25 (43%)	20 (42%)	17 (43%)	24 (40%)	28 (38%)	12 (25%)	5 (23%)	1 (50%)	**0.039**
Fatigue	2 (67%)	15 (37%)	13 (35%)	14 (39%)	11 (19%)	19 (28%)	17 (36%)	8 (40%)	0 (0%)	0.298

Number (percentage), Median. For calculation of percentages and statistics cases with missing values were excluded

^a^Frequencies of other symptoms (abdominal pain, arthralgia, pruritus, ascites, upper GI bleed, fever and nausea were too low to reliably asses differences in incidence across age categories

Bold is significant

Seventy-three patients (20%) were 60 years of age or older (≥60 group or elderly group) and 286 patients (80%) were younger than 60 years of age (<60 group or younger group).

**Fig. 2:**
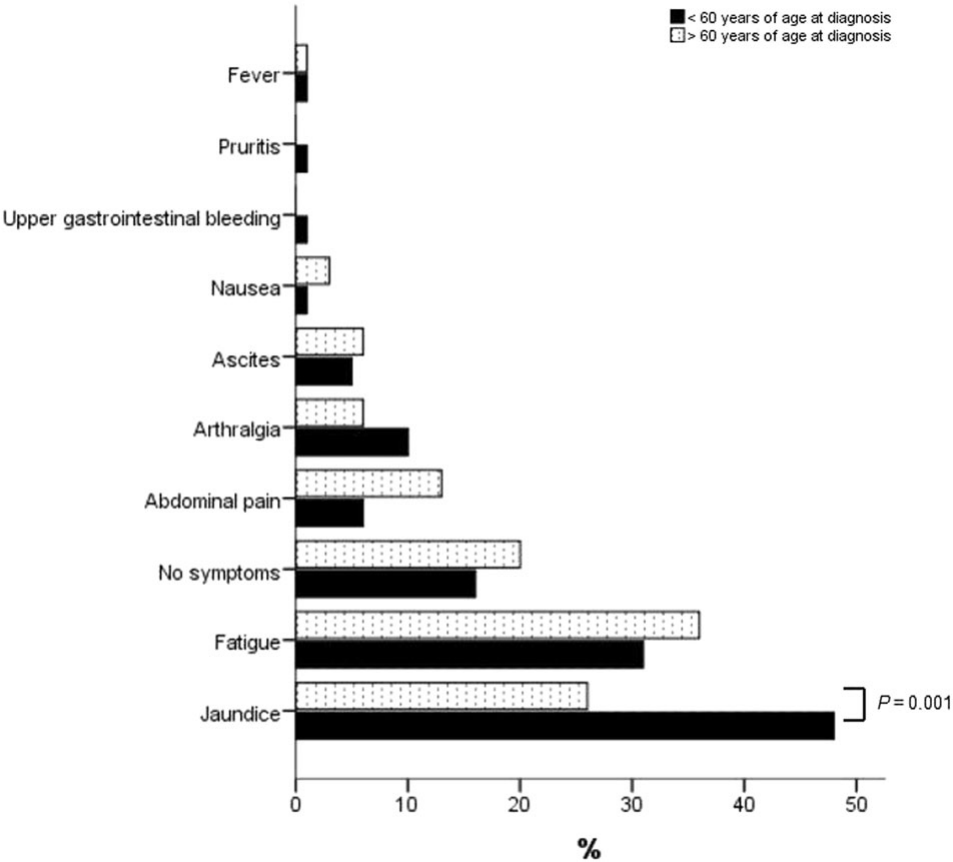
**Symptoms at AIH diagnosis up to 60 years and at or above 60 years of age**

Baseline clinical, laboratory and histological characteristics for these age categories are shown in Table 2 and symptoms at presentation in Fig. 2 (and with 65 years as cut-off in Supplementary Figure 1). Patients with onset at 60 years or later presented with significantly lower serum ALAT levels (430 vs 670 IU/l, *p* < 0.001) and more concurrent autoimmune disease (33% vs 20%, *p* < 0.05).

**Table 2:** Clinical, laboratory and histological characteristics at diagnosis

	<60 group (*N* = 286)	≥60 group (*N* = 73)	*p*-value
Age at diagnosis (year)	37,5 (5–59)	66 (60–84)	
Follow up (months)	108 (1–516)	72 (2–242)	**<0.001**
Gender (male/female)	64/222	15/58	0.874
AIH Score (1)	16 (10–22)	17 (11–22)	0.249
Alkaline phosphatase (IU/l)	154 (27–2197)	140,5 (56–391)	0.154
Alanine aminotransferase (IU/l)	442 (13–3478)	302 (26–2272)	**0.004**
IgG (g/l)	22,9 (8,16–75)	23,4 (8,19–60,7)	0.278
ANA positive	166/246 (68%)	51/71 (72%)	0.563
SMA positive	150/240 (63%)	44/68 (65%)	0.778
AMA positive	13/249 (5%)	3/72 (4%)	1.000
SLA positive	13/250 (5%)	4/71 (6%)	1.000
p-ANCA positive	42/250 (17%)	13/71 (18%)	0.725
Cirrhosis at diagnosis	81 (28%)	25 (34%)	0.310
Concurrent autoimmune disease	57 (20%)	24 (33%)	**0.027**
HLA typing	(*N* = 144)	(*N* = 28)	
HLA DR3	88 (61%)	15 (54%)	0.529
HLA DR4	35 (24%)	9 (32%)	0.477
Histological features	(*N* = 249)	(*N* = 65)	
Interface hepatitis	228 (92%)	59 (91%)	0.806
Plasma cell infiltrate	248 (99%)	64 (99%)	0.372
Biliary changes	16 (6%)	9 (14%)	0.068
Mode of presentation	(*N* = 246)	(*N* = 70)	**0.034**
Asymptomatic	38 (15%)	14 (20%)	**0.365**
Insidious	151 (61%)	49 (70%)	0.187
Acute	**57 (23%)**	**7 (10%)**	0.016

Median (range), Number (percentage), Number/Number known or measured (percentage)

Bold is significant

In the group with onset above 60 years of age 24 patients (33%) had a concurrent autoimmune disorder including thyroid disease (*n* = 12), coeliac disease (*n* = 2), ulcerative colitis (*n* = 2), arthritis (*n* = 2), Sjögren’s syndrome (*n* = 2), scleroderma (*n* = 2), systemic lupus erythematosus (*n* = 1), type one diabetes (*n* = 1), Guillain Barré syndrome (*n* = 1) and Crohn’s disease (*n* = 1). Two patients with onset above 60 were diagnosed with two concurrent autoimmune diseases. Below 60 years of age 57 patients (20%) had a concurrent autoimmune disorder including thyroid disease (*n* = 27), ulcerative colitis (n = 8), systemic lupus erythematosus (*n* = 6), type one diabetes (*n* = 4), coeliac disease (*n* = 2), Crohn’s disease (*n* = 2), sarcoidosis (*n* = 2), unclassified connective tissue disease (*n* = 2), arthritis (*n* = 1), haemolysis (*n* = 1), Sjögren’s syndrome (*n* = 1), Henoch Schönlein purpura (*n* = 1), idiopathic thrombocytopenia (*n* = 1), multiple sclerosis (*n* = 1) and myasthenia gravis (*n* = 1). Three patients with onset before age 60 were diagnosed with two concurrent autoimmune diseases. The patients above 60 significantly less often had an acute mode of presentation (10.0% vs 23.2%, *p* = 0.016) (Table 2). There were similar rates of insidious (70.0% vs 61.4%, *p* = 0.187) and asymptomatic presentation (20.0% vs 15.4%, *p* = 0.365) above and below 60 years of age at onset.

There were no other significant baseline differences in presentation of AIH related to age. There also was no difference in percentage of patients with cirrhosis at diagnosis of AIH across ages. There were no differences in lead-time (time before referral while there was suspected liver disease) (e.g. in patients with age of onset below and above 60; *p* = 0.637).

### Treatment, remission and side effects

**Fig. 3:**
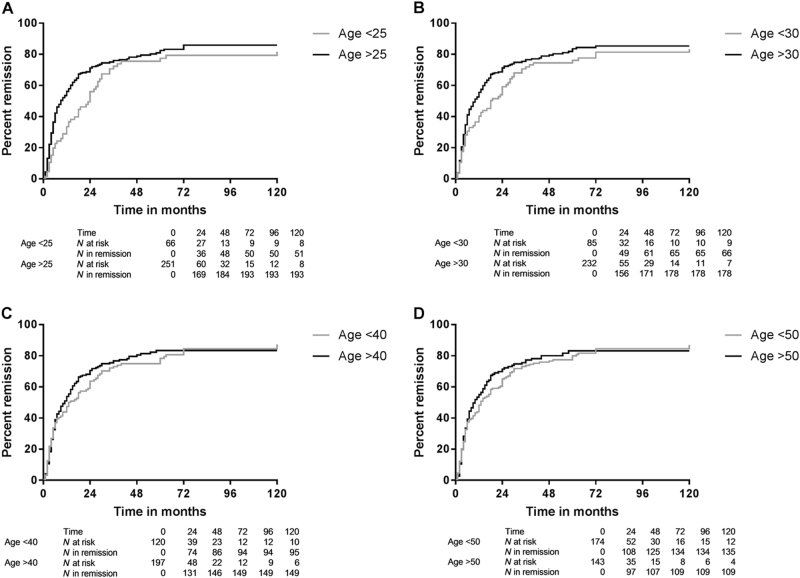
**Remission over time with age below vs at/after age.** **a** 25 (*p* = 0.022), **b** 30 (*p* = 0.089), **c** 40 (*p* = 0.619) or **d** 50 years at AIH diagnosis (*p* = 0.618)

Details on treatment effects are shown in Table 3. A mean of 80.2% of patients reached remission and 18.7% incomplete remission, with overall no differences between categories of age at AIH presentation. There were only four cases (1.1% of patients) of treatment failure, all with age on presentation between 50 and 65 years of age. In 287 patients both response to therapy and time to remission after diagnosis were known. With KM survival analysis (censored for loss to follow up, death or liver transplantation) there was less remission in patients with AIH diagnosis before age 25 than at/after age 25 years (*p* = 0.005) (Fig. 3a). There was a similar trend with age cut-off at 30 years (*p* = 0.089) (Fig. 3b), while there was no difference with age cut-off at 40 years (*p* = 0.619) (Fig. 3c), at 50 years (*p* = 0.618) (Fig. 3d), at 60 years (*p* = 0.981) (Supplementary Figure 2A) or 65 years (*p* = 0.842) (Supplementary Figure 2B). With cirrhosis at AIH diagnosis there was less remission than without cirrhosis (*p* < 0.001). As a continuous variable age at diagnosis was not a predictor of remission (*p* = 0.410). While age at AIH diagnosis below 25 years was a predictor of less remission in univariate analysis (exp(B) = 0.706, 95%CI 0.519–0.961, *p* = 0.027), in multivariate analysis it was not a predictor that was independent (exp(B) = 0.743, 95% CI 0.546–1.011, *p* = 0.059) from absence of cirrhosis at diagnosis which was a significant predictor of remission (exp(B) = 1.807, 95%CI 1.350–2.419, *p* < 0.001).

**Table 3:** Treatment effects per age category

	Age on diagnosis (year)									
	0–9 (*N* = 7)	10–19 (*N* = 58)	20–29 (*N* = 48)	30–39 (*N* = 40)	40–49 (*N* = 60)	50–59 (*N* = 73)	60–69 (*N* = 49)	70–79 (*N* = 21)	80–89 (*N* = 2)	*p*-value
Remission	6 (86%)	52 (90%)	37 (77%)	35 (88%)	46 (77%)	56 (77%)	39 (80%)	15 (71%)	1 (50%)	0.411
Incomplete response	1 (14%)	6 (10%)	11 (23%)	5 (14%)	14 (23%)	14 (19%)	9 (18%)	6 (29%)	1 (50%)	0.452
Treatment failure	0 (0%)	0 (0%)	0 (0%)	0 (0%)	0 (0%)	3 (4%)	1 (2%)	0 (0%)	0 (0%)	0.346^a^
Time to remission (months)	28	18	5	5	7.5	6	7	6	44	0.074
Loss of remission	3 (50%)	36 (71%)	21 (60%)	24 (69%)	25 (56%)	29 (52%)	22 (56%)	10 (100%)	1 (60%)	0.558
Relapse	2 (33%)	29 (56%)	20 (56%)	14 (40%)	14 (31%)	21 (38%)	15 (39%)	6 (40%)	0 (0%)	0.228
Side effects corticosteroids										
Osteoporosis	0 (0%)	7 (12%)	4 (8%)	3 (8%)	5 (8%)	16 (22%)	1 (2%)	4 (19%)	0 (0%)	**0.035**
Cushingoid changes	0 (0%)	8 (14%)	4 (8%)	8 (20%)	3 (5%)	6 (8%)	3 (6%)	1 (5%)	1 (0%)	0.239
Steroid induced diabetes	1 (14%)	2 (3%)	2 (4%)	2 (5%)	2 (3%)	6 (8%)	8 (16%)	4 (19%)	0 (0%)	0.078
Side effects immunomodulator										
Leucopenia	1 (14%)	6 (10%)	2 (4%)	1 (3%)	0 (0%)	3 (4%)	3 (6%)	0 (0%)	0 (0%)	0.210
GI-symptoms	0 (0%)	0 (0%)	2 (4%)	3 (8%)	2 (3%)	5 (7%)	2 (4%)	0 (0%)	0 (0%)	0.570

Number (percentage), Median. For calculation of percentages and statistics cases with missing values were excluded

^a^Should be interpreted with caution because of low numbers of events

Loss of remission occurred in mean 60.4% (range 50–100%) of patients and was independent from age at AIH onset. Relapse occurred in mean 42.5% (range 33.3–55.8%) of patients and was also independent from age at AIH onset.

Treatment details in patients with onset above and below 60 years of age are shown in Table 4. There were no significant differences in initial therapy, immunomodulator changes (Poisson distribution, relative risk 0.96 (95% CI 0.65–1.43)) and maintenance therapy. One hundred and forty-six patients (41%) experienced one or multiple side effects of either the prednisone, the immunomodulator or both. Ninety-six of the 359 patients (27%) developed side effects as a result of corticosteroid therapy. Diabetes was more frequent with age at AIH onset at or above 45 years vs below 45 years (10.6% vs 4.5%, *p* = 0.028). Twenty-five of the 359 patients (7%) developed side effects of the immunomodulator, mostly azathioprine, while there was no significant difference across ages.

**Table 4:** Treatment details

	<60 group (*N* = 286)	≥60 group (*N* = 72)	*p*-value
Initial therapy			0.160
Prednisone and azathioprine	235 (82%)	57 (80%)	
Prednisone	29 (10%)	5 (7%)	
No medication	7 (2%)	5 (7%)	
Budesonide and azathioprine	5 (2%)	1 (1%)	
Budesonide	4 (2%)	0 (0%)	
Other^a^	6 (2%)	4 (5%)	
Maintenance therapy			0.208
Prednisone and azathioprine	99 (35%)	19 (26%)	
Azathioprine	57 (20%)	18 (25%)	
No medication	27 (9%)	11 (15%)	
Prednisone	23 (8%)	7 (10%)	
Budesonide and azathioprine	17 (6%)	3 (4%)	
Other^b^	63 (22%)	14 (20%)	
Side effects	118 (41%)	28 (39%)	0.789
Corticosteroids			
Osteoporosis	35 (12%)	5 (7%)	0.294
Cushingoid changes	29 (10%)	4 (6%)	0.360
Steroid induced diabetes	15 (5%)	12 (16%)	**0.004**
Immunomodulator			
Leucopenia	13 (5%)	3 (4%)	1.000
Gastro-intestinal symptoms	12 (4%)	2 (3%)	0.744
Other^c^	18 (6%)	4 (6%)	

Number (percentage)

^a^Prednisone and 6-mercapopurine, ursdeoxycholic acid, prednisone and azathioprine and ursochol, prednisone and ursodeoxycholic acid, infliximab, azathioprine.

^b^23 combinations of mycophenolat mofetil, budesonide, 6-mercaptopurine, thioguanine, cyclosporine, ursodeoxycholic acid, prednisone, tacrolimus and azathioprine.

^c^Hair loss, arthralgia, liver enzyme elevations and rash.

Bold is significant

In the group with onset at/above vs below 60 years there were no differences in rates of remission, incomplete response and treatment failure (Table 5). Despite the absence of differences in loss of remission across age categories, corrected for follow-up time the patients with onset below 60 experienced significantly less loss of remission than those with onset above 60 years of age (Poisson distribution, relative risk 1.38 (95% CI 1.05–1.82, *p* = 0.022)). There was no significant difference in relapse rate after remission in patients with onset below or above 60 years of age (Poisson distribution, relative risk 1.2 (95% CI 0.78–1.86)).

**Table 5:** Outcome regarding response to treatment at the end of follow up of all AIH patients up to 60 years of age vs 60 years of age and above

	<60 group (*N* = 286)	≥60 group (*N* = 73)	*p*-value
Remission	232 (81%)	55 (76%)	0.368
Incomplete response	51 (18%)	16 (22%)	0.393
Treatment failure	3 (1%)	1 (1%)	0.806

Number (percentage). Overall *p* = 0.666

### Progression of disease

**Fig. 4:**
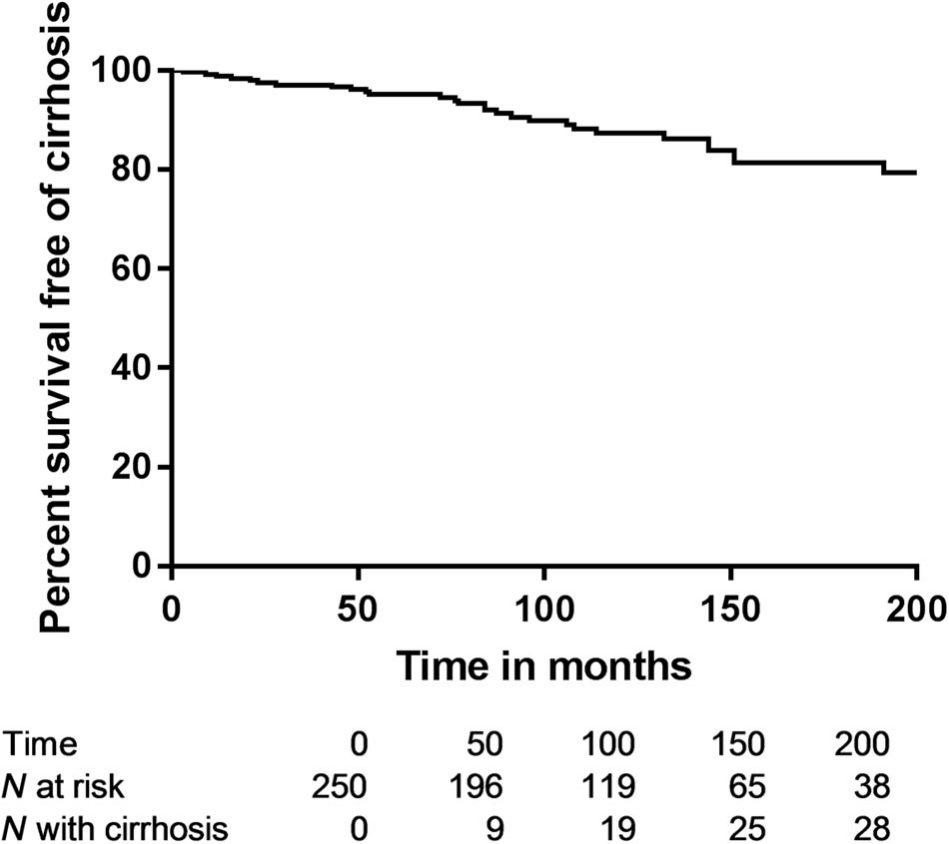
**Survival free of cirrhosis in those without cirrhosis at AIH diagnosis**

Details on progression of disease across ages at onset are shown in Table 6: progression to cirrhosis seemed to occur more frequently with AIH onset before age 30 than at or above 30 years of age (13.3 vs 6.6%. *p* = 0.036). However, correcting for differences in follow-up time with Kaplan–Meier survival analysis there was no such difference: patients before age 30 vs those at or after age 30 at AIH diagnosis remained free of cirrhosis in 86.6% vs 85.8% of cases in 160 months from diagnosis (*p* = 0.533). With other cut-offs for age at AIH diagnosis with KM analysis there also was no significant difference in rate of developing cirrhosis (with age 40 *p* = 0.983; with age 50 *p* = 0.963; with age 60 *p* = 0.607; with age 65 *p* = 0.104). The percentage of patients without cirrhosis at AIH diagnosis remaining free of cirrhosis at 1/2, 1, 2, 3, 4, 5, 10, and 20 years during follow up after AIH diagnosis was 99.7% (SE 0.3%), 99.1% (SE 0.5%), 98.2% (0.7%), 97.9% (SE 0.8%), 97.2% (0.9%), 96.5% (1.1%), 90.7% (2.0%), and 85.1% (2.9%), respectively (Fig. 4).

**Table 6:** Progression per age category

	Age on diagnosis (year)									
	0–9 (*N* = 7)	10–19 (*N* = 58)	20–29 (*N* = 48)	30–39 (*N* = 40)	40–49 (*N* = 60)	50–59 (*N* = 73)	60–69 (*N* = 49)	70–79 (*N* = 22)	80–89 (*N* = 2)	*p*-value
Time to progression (months)	191	108	105	104	92	76	79	62	54	**<0.001**
Progression	3 (43%)	21 (36%)	18 (38%)	8 (20%)	14 (23%)	11 (15%)	10 (21%)	6 (29%)	1 (50%)	0.072
Progression to										
Cirrhosis	1 (14%)	9 (16%)	5 (10%)	2 (5%)	5 (8%)	4 (6%)	2 (4%)	3 (14%)	0 (0%)	0.456
Decompensated cirrhosis	2 (29%)	5 (9%)	9 (19%)	2 (5%)	3 (5%)	2 (3%)	4 (8%)	2 (10%)	0 (0%)	**0.049**
Liver transplantation	0 (0%)	4 (7%)	3 (6%)	3 (8%)	4 (7%)	1 (1%)	1 (2%)	0 (0%)	0 (0%)	0.606
Liver-related death	0 (0%)	2 (3%)	1 (2%)	1 (2%)	1 (2%)	5 (7%)	3 (6%)	0 (0%)	1 (50%)	**0.004**
Death	0 (0%)	3 (5%)	2 (4%)	1 (3%)	2 (3%)	5 (7%)	3 (6%)	1 (5%)	1 (50%)	0.235

Number (percentage), Median. For calculation of percentages and statistics cases with missing values were excluded

Bold is significant

**Fig. 5:**
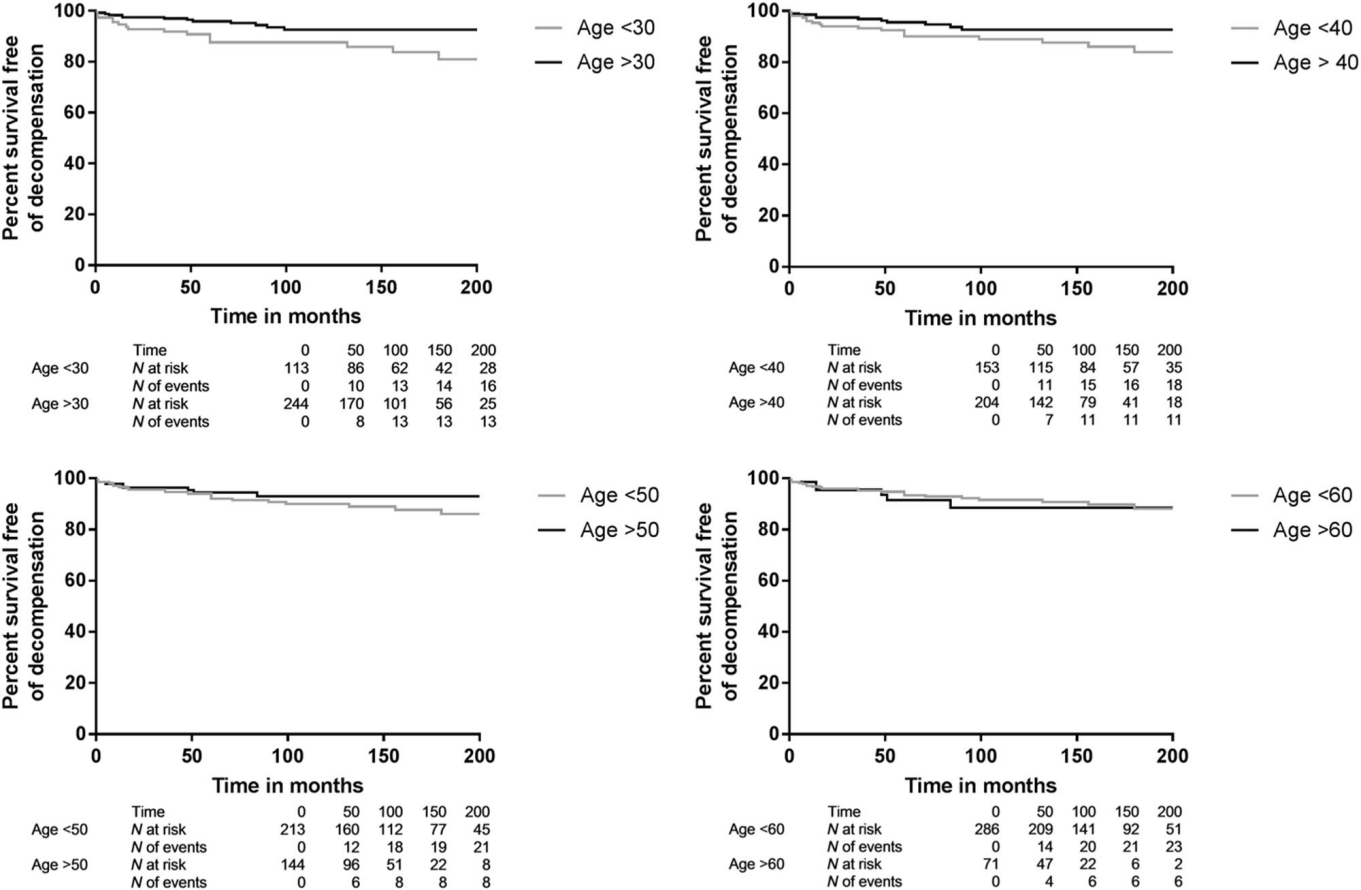
**Survival free of decompensated cirrhosis for patients with AIH diagnosis before vs at/after age** **a**. 30 (*p* = 0.02), **b** 40 (*p* = 0.09), **c** 50 (*p* = 0.32) or **d** 60 years (*p* = 0.26)

Progression to decompensated cirrhosis was more frequent with age at onset of AIH below vs at/above 30 years (*p* = 0.02), while there was no difference with AIH onset below vs at/above ages 40 (*p* = 0.09), 50 (*p* = 0.32), or 60 years (*p* = 0.61) (Fig. 5a–d).

Survival free of progression to all (combined liver-related or unrelated) death or liver transplantation was not different in KM survival analysis with AIH onset before or at/after 30 (87.5 vs 90.0% at 384 months, *p* = 0.413), 40 (86.3 vs 90.8% at 152 months, *p* = 0.994), 50 (91.9% vs 89.2% at 144 months, *p* = 0.853), or 60 years of age (90.0 vs 85.8% at 144 months, *p* = 0.809).

**Fig. 6:**
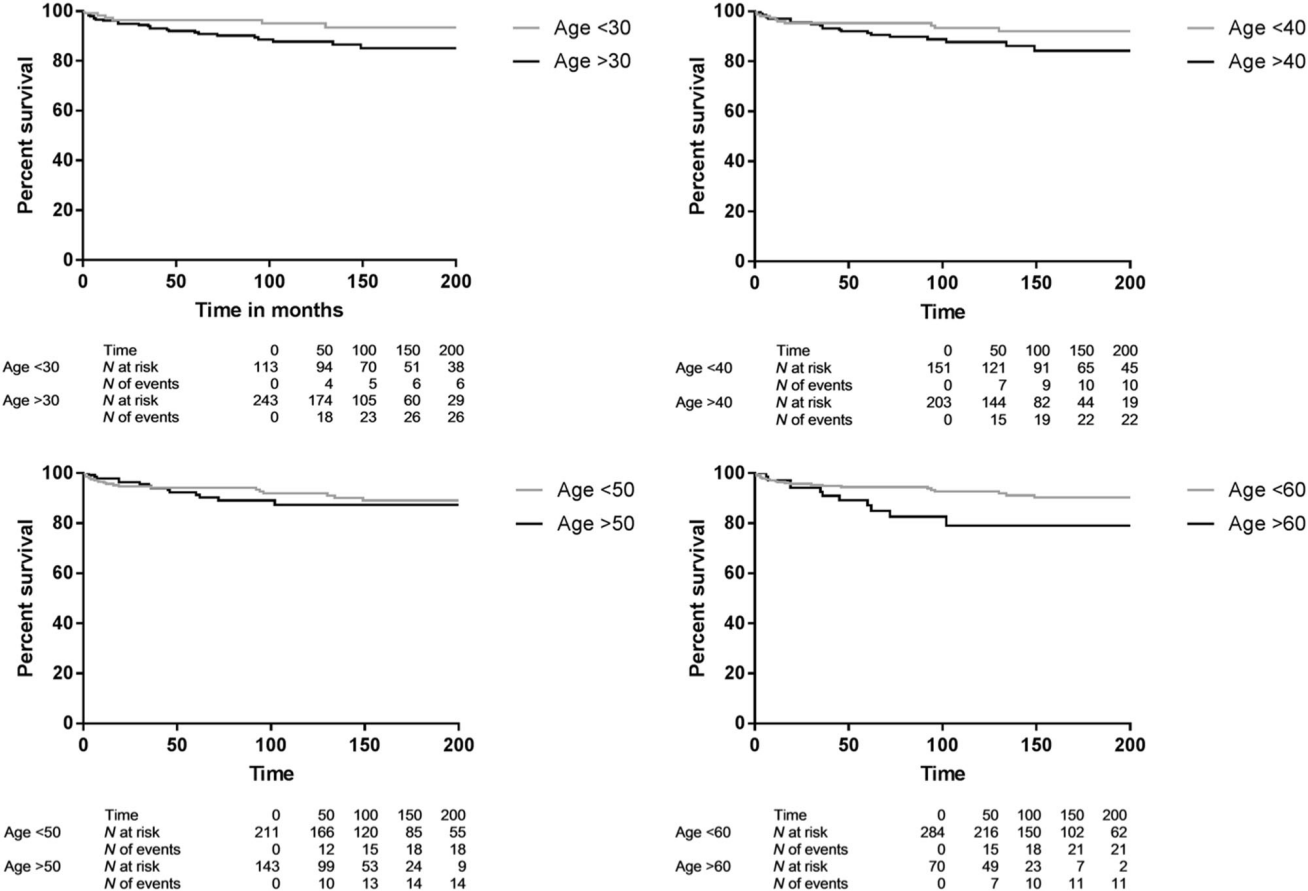
**Survival free of liver-related death or liver transplantation for those with AIH diagnosis before vs at/after age.** **a** 30 (*p* = 0.0019), **b** 40 (*p* = 0.026), **c** 50 (*p* = 0.447) or **d** 60 years (*p* = 0.012)

Based on Table 6 there appears to be more liver-related death with AIH onset at or above 45 years vs below 45 years of age (5.6% vs 2.2%, *p* = 0.004), while liver transplantation was more frequent with AIH onset below 45 years of age vs with onset at or above 45 years of age (6.7% vs 2.2%, *p* = 0.042). Correcting for follow-up time with KM survival analysis survival free of liver-related death or liver transplantation was higher for patients with AIH diagnosis before than at/after 30 years of age: (*p* = 0.019) (Fig. 6a) or 40 years of age (*p* = 0.026) (Fig. 6b). Survival free of liver-related death or liver transplantation was similar with age below 50 vs at/above 50 years at diagnosis (*p* = 0.447) (Fig. 6c), but higher with age below 60 vs at/above 60 years at diagnosis (*p* = 0.012) (Fig. 6d), or 65 years at diagnosis (*p* = 0.004) (Supplementary Figure 3). So, except below and at/above 50 years, with higher age at AIH diagnosis there was more liver-related death or liver transplantation.

As a continuous variable both age at diagnosis of AIH (exp(B) = 1.026, 95%CI 1.008–1.045, *p* = 0.006) and cirrhosis at diagnosis (exp(B) = 3.266, 95%CI 1.677–6.362, *p* = 0.001) were independent predictors of liver-related death or liver transplantation.

Time to progression overall (to cirrhosis, to decompensated cirrhosis, liver transplantation or liver-related death) appears shorter with increasing age on diagnosis of AIH across age categories (*p* < 0.001) (Table 6). However, correcting for follow-up time with Cox regression analysis age at AIH diagnosis was not related to time to disease progression as defined overall and in subgroups (no cirrhosis at diagnosis: HR 0.99 (95% CI 0.97–1.01, *p* = 0.220), with cirrhosis at diagnosis: HR 1.01 (95% CI 0.99–1.03) and with decompensated cirrhosis at diagnosis: HR 1.01 (95% CI 0.98–1.05)).

Outcome below vs at or above age 60 is shown in Table 7: there were no significant differences between these age groups, although there was only one (1.4%) liver transplantation with onset of AIH above age 60 vs 15 (5.2%) with onset below 60 years (*p* = 0.162).

**Table 7:** Rates of disease progression at the end of follow up with age below 60 vs at/above 60 years on diagnosis

	<60 group	≥60 group	p-value
No cirrhosis at diagnosis	(*N* = 203)	(*N* = 46)	0.234
No progression	163 (80%)	37 (81%)	
Progression to compensated cirrhosis	25 (12%)	5 (11%)	
Progression to decompensated cirrhosis	12 (6%)	2 (4%)	
Progression to liver transplant	3 (1%)	0 (0%)	
Progression to liver-related death	2 (1%)	2 (4%)	
Compensated cirrhosis at diagnosis	(*N* = 52)	(*N* = 12)	0.607
No progression	30 (57%)	8 (67%)	
Progression to decompensated cirrhosis	11 (21%)	4 (33%)	
Progression to liver transplant	7 (14%)	0 (0%)	
Progression to liver-related death	4 (8%)	0 (0%)	
Decompensated cirrhosis at diagnosis	(*N* = 29)	(*N* = 13)	0.717
No progression	18 (62%)	9 (69%)	
Progression to liver transplant	5 (17%)	1 (8%)	
Progression to liver-related death	6 (21%)	3 (23%)	

Number (percentage)

Nine patients never started medication, five patients at/above 60 and four below age 60 years. Despite the lack of treatment, four of them reached remission (among which one elderly patient), the other five had an incomplete response (among which four elderly patients). At the end of follow up 11 patients above 60 years and 27 patients below age 60 at diagnosis received no treatment (including the nine previously mentioned patients). Outcome of these patients is shown in Table 8. Reasons for stopping treatment were unknown for all patients.

**Table 8:** Outcome at the end of follow up of untreated AIH patients

	<60 group (*N* = 27)	60 group (*N* = 11)	*p*-value
Follow up (months)	89 (12–444)	57 (8–118)	0.082
Remission	22 (81%)	7 (64%)	0.627
Incomplete response	4 (15%)	4 (36%)	0.058
Treatment failure	1 (4%)	0	0.564
Disease progression			0.311
No progression	21 (78%)	10 (91%)	
To compensated cirrhosis	0 (0%)	0 (0%)	
To decompensated cirrhosis	2 (7%)	0 (0%)	
To liver transplant	2 (7%)	0 (0%)	
To liver-related death	1 (4%)	0 (0%)	
Unknown	1 (4%)	1 (9%)	

Number (percentage)

Analysis with 65 years as age cut-off yielded similar results as with age 60, as shown in Supplementary Tables 1–4 and Supplementary Figures 1–3.

## Discussion

### Presentation of AIH

There were no significant differences across age categories in mode (acute, insidious or asymptomatic) of presentation. ALAT was highest with AIH onset between 30 and 60 years of age. INR was higher with onset below 20 years and between 40 and 50 years. As in other studies the incidence of HLA-DR4 was higher with age at diagnosis at or above 40 years, and there was a bimodal pattern in age at diagnosis, with one peak in the second and one in the fifth decade^6,10,16,21^. The patients included in this study originated from four academic centres in contrast to some reports from non-academic centres, where one age-peak between the fourth and seventh decade was seen^5,6,8–11^. The finding that one in five patients were at or above the age of 60 at diagnosis, confirms a recent meta-analysis of smaller studies^16^. The finding that patients above 60 present with lower serum alanine aminotransaminase levels and with less jaundice than younger patients is in concurrence with most previous studies, although three studies found no difference in mode of onset. There could be a referral bias, as the sickest younger patients may more often than elderly patients have been transferred to tertiary referral centres because of their expertise and possibility of liver transplantation^6,8,9,12,13^. There were significantly more autoimmune diseases with onset above 60 years as compared to younger patients, with thyroid diseases by far being the most frequent in both groups. Previous studies did not find significant differences in concurrent autoimmune diseases between younger and elderly patients, but the studies by Granito et al. and Czaja et al. did show a trend towards more autoimmune diseases in elderly. It is possible that in the previous studies significance was not reached because of small sample sizes^6,10,16^. In a recently performed meta-analysis it was concluded that patients aged above 60 or 65 present more often with cirrhosis at diagnosis^16^. Our data does not support these findings, as the percentage of cirrhosis at diagnosis was around 30% at all ages. Taking a detailed look at the meta-analysis, six out of the nine studies found no difference in cirrhosis at diagnosis between young and elderly patients. We studied whether there was a difference between age groups in time before referral while there already was suspected liver disease, but there was no such lead-time bias^5,6,8–14^.

### Treatment, remission

There were no differences in initial and maintenance therapies between younger and elderly patients in the current data. Treatment was equally tolerated in all age groups. The 27% side effects as a result of corticosteroid therapy in the current study was lower than in previous reports that mention corticosteroid-related side effects in as many as 80% of patients^19^. Most studies are retrospective studies, which can lead to over- or underreporting and different definition of side effects. Diabetes was more frequent with age at AIH onset at or above 45 years, which indicates that elderly patients may benefit more from corticosteroid-sparing maintenance options. The 7% side effects from the immunomodulator, mostly azathioprine, was comparable to previous reports, with no significant difference between elderly and younger patients^19^.

Of all patients 80% (71–90%) reached remission and 19% (10–29%) incomplete remission, with treatment failure in only four (1%) patients aged 50–65 years at onset. Age was not an independent predictor of remission, while cirrhosis at diagnosis was. The overall response to treatment was comparable to previous reports^8–11,22^. In contrast to previous reports there were overall no differences in rates of relapse or loss of remission, except for patients with onset at or above age 60 who—when corrected for follow-up time—experienced more loss of remission^8,9,16^. Fear of more side effects may have led to suboptimal treatment and more rapid tapering of medication in some patients with diagnosis over age 60^8,10^. Unfortunately, exact treatment and dosing schedules were not available for all patients to evaluate treatment schedules and alternative therapies in more detail^23^.

### Progression

Lower age at AIH diagnosis and cirrhosis were independent predictors of survival without liver-related death or liver transplantation. This was despite the finding that development of decompensated cirrhosis was more common with AIH onset below an age of 30 years. In patients without cirrhosis on diagnosis there was a linear progression towards cirrhosis over time, which was 10% in the first 10 years, and in KM analysis age at diagnosis did not influence this rate. Despite the fact that the majority of patients reaches remission, and survival without liver-related death or liver transplantation is quite good, disease progression despite treatment occurs and is an important target for future research. This may be due to continuing inflammation, which can be present in liver biopsies despite biochemical remission^24^. On the other hand the advantage of complete over incomplete remission is debatable, since in a previous study survival with incomplete remission did not differ from patients with complete biochemical remission^25^.

The age influence on presentation and on survival free of liver-related death or liver transplantation and the absent influence of age on remission are novel findings not mentioned in earlier reports^25–27^. Nevertheless, this study carries the limitations of a—partially—retrospective study with some missing values. Data beyond ten years after diagnosis may be less accurate, since prospective inclusion of patients in the current cohort started in 2006. Strengths are the large cohort of patients with long-term follow up, the detailed analysis of presenting signs and symptoms, and the first unbiased analysis of the role of age in presentation, response to therapy and disease progression.

These data support the idea that at all ages in patients with liver disease AIH should be seriously considered, and that treatment of AIH should be according to the current guidelines at all ages, while recognising the observed differences between elderly and younger patients during maintenance therapy^28^.

## Study Highlights

### What is current knowledge?

- Autoimmune hepatitis is an important cause of liver disease in elderly patients as 20%–25% of patients are above the age of 60 at diagnosis.
- Ten studies with relatively small sample size have specifically addressed AIH in elderly patients and have yielded diverging results with respect to baseline clinical, laboratory and liver histological characteristics, mode of presentation, response to treatment and long-term outcome.
- In a meta-analysis of these studies elderly patients were more likely to be cirrhotic and asymptomatic at diagnosis, had the same response to treatment as compared to younger patients, but were less likely to relapse after withdrawal of treatment

### What is new here?

- The role of age was studied unbiasedly.
- The rate of cirrhosis around 30% at diagnosis of AIH was similar at all ages.
- ALAT levels were higher in patients aged 30–60 years on AIH diagnosis.
- Patients with AIH diagnosis above age 60 less frequently had an acute onset, had lower alanine aminotransaminase levels, less jaundice and more concurrent autoimmune disease, but a comparable rate of cirrhosis as compared to younger patients.
- In contrast to cirrhosis, age at AIH diagnosis was not an independent predictor of remission.
- Remission was reached in 80.2%, incomplete remission in 18.7%, only 1.1% (all aged 50–65) was treatment-refractory.
- Overall loss of remission and relapse were independent from age at AIH diagnosis, but with AIH onset above age 60 there was more loss of remission
- Diabetes was more frequent in patients diagnosed after age 45 years.
- Age was not related to the risk of progression to cirrhosis, which is 10% in 10 years
- If AIH was diagnosed before age 30 more decompensated cirrhosis developed
- Higher age at AIH diagnosis and cirrhosis were independent predictors of lower survival free of progression to liver-related death or liver transplantation

## Electronic supplementary material

Supplemental Figure 1

Supplemental Figure 2

Supplemental Figure 3

Supplemental Table 1

Supplemental Table 2

Supplemental Table 3

Supplemental Table 4

## References

[1] Alvarez F (1999). International Autoimmune Hepatitis Group Report: review of criteria for diagnosis of autoimmune hepatitis. J. Hepatol..

[2] Zachou K (2013). Review article: autoimmune hepatitis—current management and challenges. Aliment. Pharmacol. Ther..

[3] Bearn AG, Kunkel HG, Slater RJ (1956). The problem of chronic liver disease in young women. Am. J. Med..

[4] Bartholomew LG, Hagedorn AB, Cain JC, Baggenstoss AH (1958). Hepatitis and cirrhosis in women with positive clot tests for lupus erythematosus. N. Engl. J. Med..

[5] Verslype C (2005). Diagnosis and treatment of autoimmune hepatitis at age 65 and older. Aliment. Pharmacol. Ther..

[6] Granito A (2005). Clinical features of type 1 autoimmune hepatitis in elderly Italian patients. Aliment. Pharmacol. Ther..

[7] McFarlane IG (1998). The relationship between autoimmune markers and different clinical syndromes in autoimmune hepatitis. Gut.

[8] Al-Chalabi T, Boccato S, Portmann BC, McFarlane IG, Heneghan MA (2006). Autoimmune hepatitis (AIH) in the elderly: a systematic retrospective analysis of a large group of consecutive patients with definite AIH followed at a tertiary referral centre. J. Hepatol..

[9] Schramm C, Kanzler S, zum Buschenfelde KH, Galle PR, Lohse AW (2001). Autoimmune hepatitis in the elderly. Am. J. Gastroenterol..

[10] Czaja AJ, Carpenter HA (2006). Distinctive clinical phenotype and treatment outcome of type 1 autoimmune hepatitis in the elderly. Hepatology.

[11] Parker DR, Kingham JG (1997). Type I autoimmune hepatitis is primarily a disease of later life. QJM.

[12] Miyake Y (2007). Clinical features of Japanese elderly patients with type 1 autoimmune hepatitis. Intern. Med..

[13] Newton JL (1997). Autoimmune hepatitis in older patients. Age Ageing.

[14] Floreani A (2006). Type I autoimmune hepatitis: clinical course and outcome in an Italian multicentre study. Aliment. Pharmacol. Ther..

[15] Xie B (2010). Autoimmune hepatitis in elderly is usually an advanced disease at presentation, is less symptomatic and relapse is infrequent. Gastroenterol.

[16] Chen J, Eslick GD, Weltman M (2014). Systematic review with meta-analysis: clinical manifestations and management of autoimmune hepatitis in the elderly. Aliment. Pharmacol. Ther..

[17] Chazouilleres O (1998). Primary biliary cirrhosis-autoimmune hepatitis overlap syndrome: clinical features and response to therapy. Hepatology.

[18] van Buuren HR, van Hoogstraten HJE, Terkivatan TF, Schalm SW, Vleggaar FP (2000). High prevalence of autoimmune hepatitis among patients with primary sclerosing cholangitis. J. Hepatol..

[19] Manns MP (2010). Diagnosis and management of autoimmune hepatitis. Hepatology.

[20] van Gerven NM (2013). Relapse is almost universal after withdrawal of immunosuppressive medication in patients with autoimmune hepatitis in remission. J. Hepatol..

[21] van Gerven NM (2015). HLA-DRB1*03:01 and HLA-DRB1*04:01 modify the presentation and outcome in autoimmune hepatitis type-1. Genes Immun..

[22] Lamers MM, van Oijen MG, Pronk M, Drenth JP (2010). Treatment options for autoimmune hepatitis: a systematic review of randomized controlled trials. J. Hepatol..

[23] Baven-Pronk AM (2011). The role of mycophenolate mofetil in the management of autoimmune hepatitis and overlap syndromes. Aliment. Pharmacol. Ther..

[24] Dhaliwal HK (2015). Long-term prognositic significance of persisting histological activity despite biochemical remission in autoimmune hepatitis. Am. J. Gastroenterol..

[25] Hoeroldt B (2011). Long-term outcomes of patients with autoimmune hepatitis managed at a nontransplant center. Gastroenterology.

[26] Werner M (2010). Characteristics and long-term outcome of patients with autoimmune hepatitis related to the initial treatment resonse. Scand. J. Gastroenterol..

[27] Feld JJ (2005). Autoimmune hepatitis: effect of symtoms and cirrhosis on natural history and outcome. Hepatology.

[28] European Association for the Study of the Liver. EASL Clinical Practice Guidelines: Autoimmune hepatitis. J. Hepatol. 63, 971–1004 (2015). 10.1016/j.jhep.2015.06.030 26341719

