# Supplementary figures and images for: Role of age in presentation, response to therapy and outcome of autoimmune hepatitis
Source: Clin Transl Gastroenterol. 2018 Jul 2;9(6):165. doi: 10.1038/s41424-018-0028-1 (PMC6026593; doi:10.1038/s41424-018-0028-1)

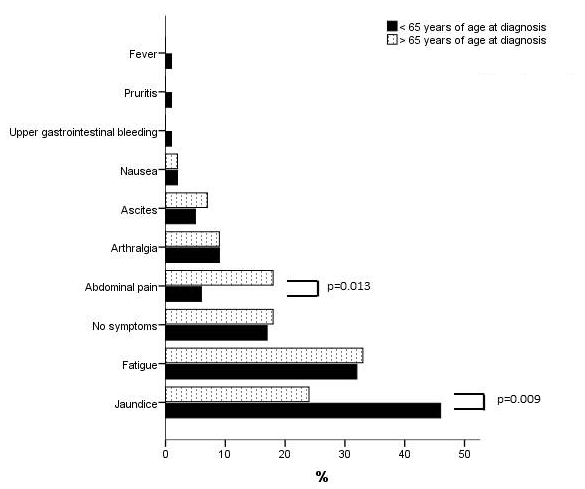

Supplement: Supplementary file 1 — Supplemental Figure 1 [file 41424_2018_28_MOESM2_ESM.tif]

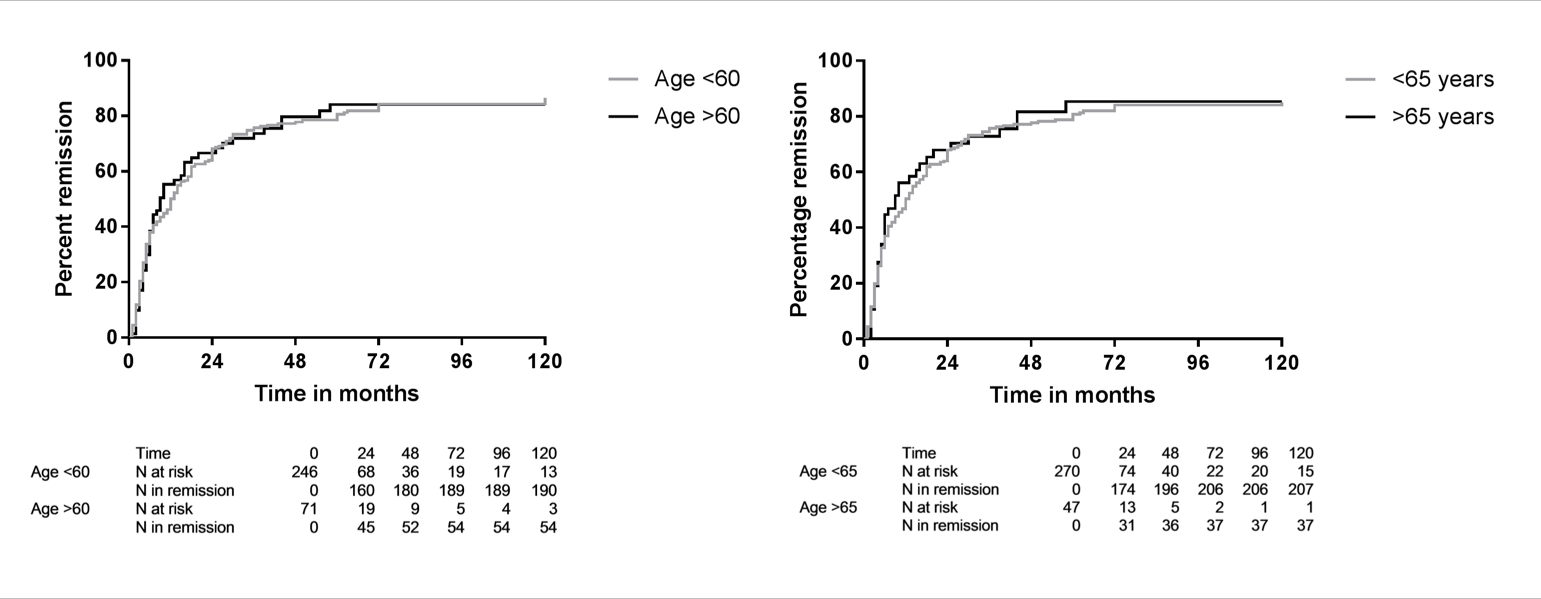

Supplement: Supplementary file 2 — Supplemental Figure 2 [file 41424_2018_28_MOESM3_ESM.tif]

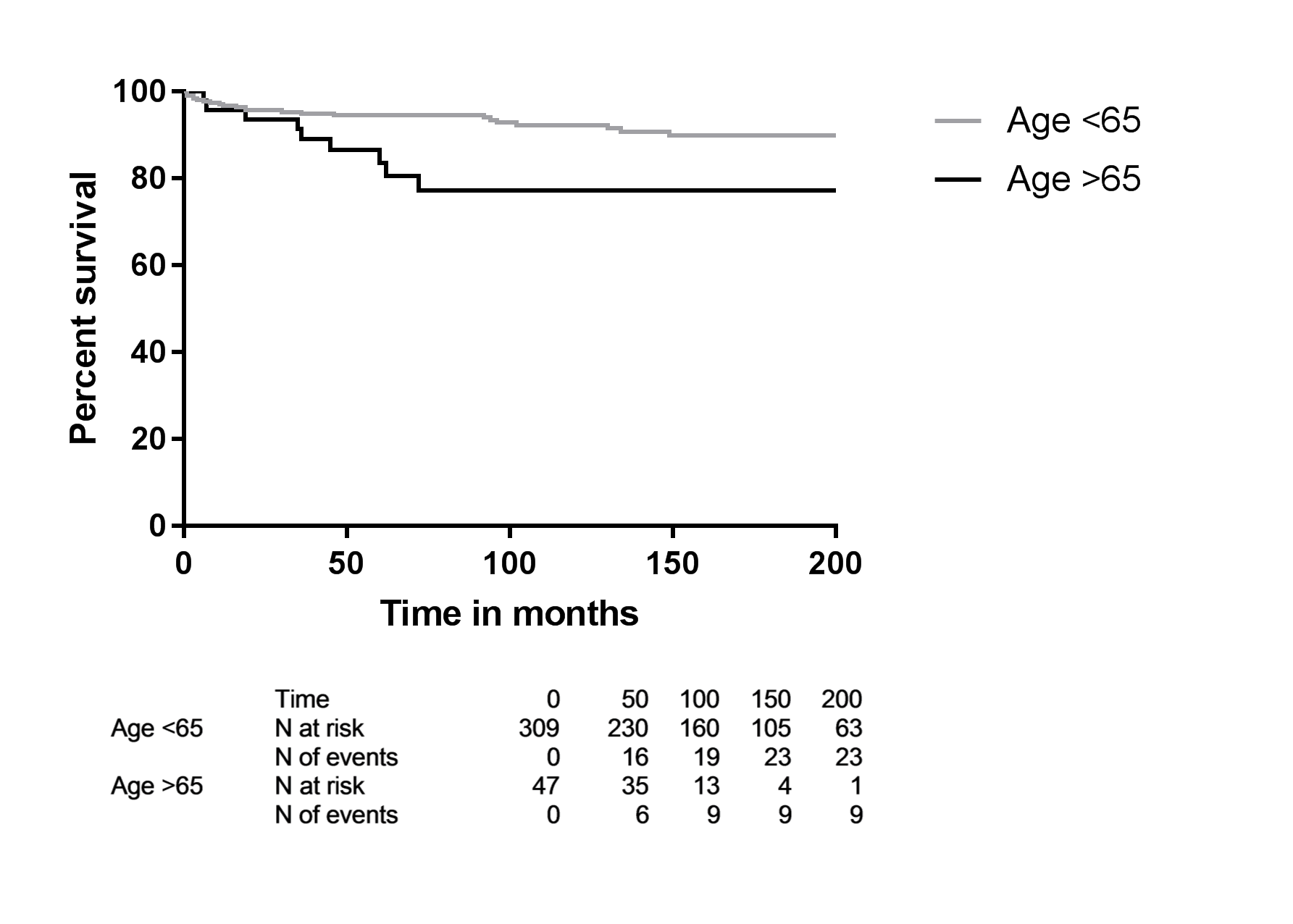

Supplement: Supplementary file 3 — Supplemental Figure 3 [file 41424_2018_28_MOESM4_ESM.tif]
