# Supplementary material for: Role of age in presentation, response to therapy and outcome of autoimmune hepatitis
Source: Clin Transl Gastroenterol. 2018 Jul 2;9(6):165. doi: 10.1038/s41424-018-0028-1 (PMC6026593; doi:10.1038/s41424-018-0028-1)
Supplement: Supplementary file 4 — Supplemental Table 1 [file 41424_2018_28_MOESM5_ESM.docx]

| **Supplementary Table 1.** Clinical, laboratory and histological characteristics at diagnosis with age 65 as cut-off | | | |
| --- | --- | --- | --- |
|  | < 65 group  (N = 311) | ≥ 65 group  (N = 48) | p-value |
| Age at diagnosis (year) | 40 (5-64 ) | 69,5 (65-84) |  |
| Follow up (months) | 108 (1-516) | 72 (6-219) | **0.002** |
| Gender (male/female) | 69/242 | 10/38 | 1.000 |
| AIH Score (1) | 16 (10-22) | 17 (11-22) | **0.037** |
| Alkaline phosphatase (IU/l) | 154 (27-2197) | 137 (63-391) | 0.182 |
| Alanine transaminase (IU/l) | 440,5 (13-3478) | 304 (37-2272) | **0.032** |
| IgG (g/l) | 22,4 (8,16-75) | 27,5 (8,19-46,7) | 0.900 |
| ANA positive | 183/271 (68%) | 34/46 (74%) | 0.493 |
| SMA positive | 163/264 (62%) | 31/44 (71%) | 0.314 |
| AMA positive | 14/274 (5%) | 2/47 (4%) | 1.000 |
| SLA positive | 14/275 (5%) | 3/46 (7%) | 0.720 |
| p-ANCA positive | 44/275 (16%) | 11/46 (24%) | 0.205 |
| Cirrhosis at diagnosis | 89 (29%) | 17 (30%) | 0.307 |
| Concurrent autoimmune disease | 64 (21%) | 17 (35%) | **0.027** |
|  | | | |
| HLA typing | (N = 152 ) | (N = 20) |  |
| HLA DR3  HLA DR4 | 94 (62%)  37 (24%) | 9 (45%)  7 (35%) | 0.156  0.291 |
|  | | | |
| Histological features | (N = 272) | (N = 42) |  |
| Interface hepatitis  Plasma cell infiltrate  Biliary changes | 249 (92%)  270 (99%)  17 (6%) | 38 (91%)  42 (100%)  8 (19%) | 0.770  1.000  **0.010** |
|  | | | |
| Mode of presentation | (N = 270 ) | (N = 46) |  |
| Asymptomatic  Insidious  Acute | 44 (16%)  165 (61%)  61 (23 %) | 8 (17%)  35 (76%)  3 (7%) | 0.087 |
| *Median (range), Number (percentage), Number/Number known or measured (percentage)* | | | |
