# Supplementary material for: Role of age in presentation, response to therapy and outcome of autoimmune hepatitis
Source: Clin Transl Gastroenterol. 2018 Jul 2;9(6):165. doi: 10.1038/s41424-018-0028-1 (PMC6026593; doi:10.1038/s41424-018-0028-1)
Supplement: Supplementary file 5 — Supplemental Table 2 [file 41424_2018_28_MOESM6_ESM.docx]

| **Supplementary Table 2.** Treatment details with 65 years of age as cut-off | | |  |
| --- | --- | --- | --- |
|  | < 65 group  (N = 311 ) | ≥ 65 group  (N = 47) | p-value |
| Initial therapy  Prednisone and azathioprine  Prednisone  No medication  Budesonide and azathioprine  Budesonide  Other * | 254 (82%)  30 (10%)  10 (3%)  5 (2%)  4 (1%)  8 (2%) | 38 (81%)  4 (9%)  2 (4%)  1 (2%)  0 (0%)  2 (4%) | 0.489 |
| Maintenance therapy  Prednisone and azathioprine  Azathioprine  No medication  Prednisone  Budesonide and azathioprine  Other ** | 101 (32%)  67 (22%)  31 (10%)  26 (8%)  18 (6%)  68 (22%) | 17 (36%)  8 (17%)  7 (15%)  4 (9%)  2 (4%)  14 (19%) | 0.677 |
| Side effects  Corticosteroids  Osteoporosis  Cushingoid changes  Steroid induced diabetes  Immunomodulator  Leucopenia  Gastro-intestinal symptoms  Other **** | 129 (42%)  36 (12%)  30 (10%)  19 (6%)  15 (5%)  13 (4%)  19 (6%) | 17 (36%)  4 (9%)  3 (6%)  8 (17%)  1 (2%)  1 (2%)  3 (6%) | 0.528  0.803  0.597  **0.015**  0.705  1.000 |
| *Number (percentage)*  ** Prednisone and 6-mercapopurine, ursdeoxycholic acid, prednisone and azathioprine and ursochol, prednisone and ursodeoxycholic acid, infliximab, azathioprine.*  *** 23 combinations of mycophenolat mofetil, budesonide, 6-mercaptopurine, thioguanine, cyclosporine, ursodeoxycholic acid, prednisone, tacrolimus and azathioprine.*  **** Hair loss, arthralgia, liver enzyme elevations and rash.* | | | |
