# Supplementary material for: Role of age in presentation, response to therapy and outcome of autoimmune hepatitis
Source: Clin Transl Gastroenterol. 2018 Jul 2;9(6):165. doi: 10.1038/s41424-018-0028-1 (PMC6026593; doi:10.1038/s41424-018-0028-1)
Supplement: Supplementary file 6 — Supplemental Table 3 [file 41424_2018_28_MOESM1_ESM.docx]

| **Supplemental Table 3.** Response to treatment at the end of follow up of all AIH patients up to 65 years of age versus 65 years of age and above. | | | |
| --- | --- | --- | --- |
|  | < 65 group  (N = 311 ) | >65 group  (N = 47) | p-value |
| Remission | 250 (80%) | 37 (79%) | 0.844 |
| Incomplete response | 57 (18%) | 10 (21%) | 0.688 |
| Treatment failure | 4 (1%) | 0 (0%) | 0.970 |
| *Number (percentage)* | | |  |
