# Supplementary material for: Role of age in presentation, response to therapy and outcome of autoimmune hepatitis
Source: Clin Transl Gastroenterol. 2018 Jul 2;9(6):165. doi: 10.1038/s41424-018-0028-1 (PMC6026593; doi:10.1038/s41424-018-0028-1)
Supplement: Supplementary file 7 — Supplemental Table 4 [file 41424_2018_28_MOESM7_ESM.docx]

| **Supplementary Table 4.** Disease progression at the end of follow up of all AIH patients up to 65 years of age versus 65 years of age and above. | | | |
| --- | --- | --- | --- |
|  | < 65 group | ≥ 65 group | p-value |
| No cirrhosis at diagnosis  No progression  Progression to compensated cirrhosis  Progression to decompensated cirrhosis  Progression to liver transplant  Progression to liver related death | (N = 221)  176 (80%)  25 (12%)  14 (6%)  3 (1%)  3 (1%) | (N = 30)  24 (80%)  5 (17%)  0 (0%)  0 (0%)  1 (3%) | 0.371 |
| Compensated cirrhosis at diagnosis  No progression  Progression to decompensated cirrhosis  Progression to liver transplant  Progression to liver related death | (N = 55)  32 (58%)  12 (22%)  7 (13%)  4 (7%) | (N = 9)  6 (67%)  3 (33%)  0 (0%)  0 (0%) | 0.875 |
| Decompensated cirrhosis at diagnosis  No progression  Progression to liver transplant  Progression to liver related death | (N = 34)  21 (62%)  6 (17%)  7 (21%) | (N = 8)  6 (75%)  0 (0%)  2 (25%) | 0.963 |
| *Number (percentage)* | | | |
